# The influences of electric field intensity and driving force on the slip behaviour of water flow in a nanochannel

**DOI:** 10.1371/journal.pone.0257589

**Published:** 2021-09-22

**Authors:** Qiwei Liu, Dezheng Wang, Miao Yu, Biao Cong, Xiaopeng Yu

**Affiliations:** 1 Jilin Provincial Key Laboratory for Numerical Simulation, Jilin Normal University, Siping, Jilin, P. R. China; 2 Beijing Institute of Mechanical and Electrical Engineering, Beijing, P. R. China; 3 School of Materials Science and Engineering, Institute for Advanced Materials, Jiangsu University, Zhenjiang, P. R. China; Tongji University, CHINA

## Abstract

In the present work, non-equilibrium molecular dynamics (MD) simulations are used to investigate the flow of liquid water between two metallic solid atomistic smooth walls. The present work focuses on the combined effect of external electric field and driving force on the slip behaviour and structure of liquid water at the solid-water interface. The upper wall of the set model is positively charged, and the lower wall of the model is negatively charged. The simulation results show that as the driving force increases, the slip length also increases. At a given driving force, no matter how the electric field intensity changes, there is almost no change in the slip length, so the slip length is independent of the electric field strength. In addition, the results found that there is a linear relationship between the slip length and the normalised main peak of the static structure factor under different driving forces.

## 1 Introduction

Since the 1990s, as the scientific community has re-studied classical theories and tried to build models of different scales to understand the complex mechanisms involved in metal/fluid interaction, the nanoscale aspects of fluid mechanics have attracted increasing attention [1]. On the macro-scale, the slip behaviour of liquids on a metal surface is often ignored, but on the micro-nano-scale, one of the remarkable features is the presence of a large surface-volume ratio, which leads to a significant influence on the slip behaviour. For example, it has a significant influence on applied research into desalination of seawater, The pressure-driven transport of saline (NaCl) water through the nanochannels formed by a graphene (GE) bilayer with and without a vertical electric field [2–4], bioengineering [5, 6], medical systems [7], *etc*.

The simulation techniques used to illustrate the influences of electric field strength and driving force on slip behaviour on the micro-nano scale is mainly conducted by molecular dynamics (MD) methods [8]. MD have been used to determine the properties of fluids and their transport in nanochannels [9–11], and many experimental researchers have begun to use MD to study the slip behaviour of nanoscale fluids on metal surfaces. The behaviour of slip has a significant impact on fluid flow at the micro-nano-scale. Experimental studies related to slip behaviour are difficult because the velocity of fluid flow on the metal surface is non-uniform [12], however, one can use MD simulation because it can predict the velocity distribution at the atomic level with no need to make any assumptions about the length of the metal wall.

In recent years, few people have studied the effects of electric field strength and driving force on the slip behaviour of fluid flow. This is mainly because such theoretical research and experiments are difficult. We use MD simulation to investigate the slip behaviour of electric field strength and driving force on fluid flow, and analyse the velocity and density curves separately through use of software. When the driving force is tangential to the wall surface, the liquid flows, and it has been found that there is a dependence between the flow characteristics and the surface charge density (CD). It should also be noted that the direction of the driving force exerts an important influence on the transport and flow characteristics of water in a nanochannel [13], so we define the direction of the driving force in the *y*-direction.

In addition to the condition of electric field strength, we also need a condition pertaining to the driving force. One of our goals is to provide a phenomenological continuous model for force-driven liquid flow in nano-channels, which can predict nanometric behaviour across a range of length-scales. Velocity distribution and volume and mass flow in the channel have also been modelled [14, 15], with each model needing to be calibrated individually under specific conditions. The model calibration step is critical because the slip length and other model parameters depend on the liquid-solid pair and the desired thermodynamic state [14]. Furthermore, It is also noted that the slip length can be used to quantify micro- or nano-level hydrophobicity [16]. Slip length is an intuitive manifestation of slip behaviour; through the change of slip length, a more intuitive sense of the manifestation of slip behaviour is realised.

The charged surface creates an additional method with which to control the micron and nano-scale characteristics of the flow [17], so it is more conducive to the present study of slip behaviour, and liquid water will also bind with the metal surface through the arc of oxygen atoms. Sofos *et al*. [18] used MD simulations to apply electric fields of various strengths to allow clean water to flow inside the channels. They found that the electric field is not used to drive the flow as in a nanoscale membrane process. Celebi *et al*. [19] also noted that the water molecules redirect the oxygen atoms to the positively charged surface and redirect the dipoles [20], however, there is no systematic study of the comprehensive effects of the interaction of electric field strength and driving force on the slip behaviour of fluid flowing on a metal surface. Therefore, the present research focussed on the effects of the electric field strength and driving force on the sliding behaviour of fluid flowing on a metal surface.

In this article, MD simulation is used to investigate the effects of various electric field strengths and driving forces on slip behaviour. Sofos *et al*., undertook MD simulations of ion separation in nano-channel water flows using an electric field [17]. Renou *et al*. used pressure-driven MD simulations of water transport through a hydrophilic nanochannel [21]. There are few reports on the effects of applying driving force and electric field strength to water in nanochannels at the same time. The main purpose of this research is to investigate the effects of electric field strength on the structure and transport properties of water on the metal surface. Aluminium is a common metal, so it is used to represent the metal material herein. We mainly focus on the density distribution, velocity distribution, slip length, electric field strength, and driving force. The simulation provides a modelling structure in the form of plots of the density and velocity distributions under various conditions.

## 2 Methods

**Fig 1:**
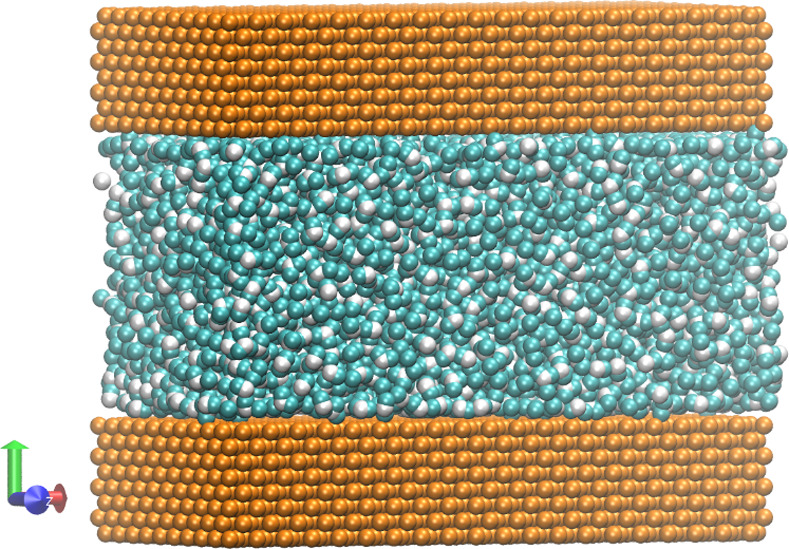
A simulation model of liquid water enclosed between two plane atomic smooth walls. Each wall is composed of 19,528 atoms. There are 4394 molecules comprising the liquid water.

The present experiment refers to the research method of Celebi *et al*. and is designed to observe the flow behaviour of liquid water on a metal surface [19]. As shown in Fig 1, the three-dimensional model is composed of water molecules between two fixed metals. The wall is composed of four sides and has a face-centred cubic structure with a lattice constant of 4.05 Å. The size of the simulation area is set to 3.69 × 3.81 × 5.44 nm in the *x*, *y*, and *z*-directions. The channel height *h* is 4.0 nm, which is large enough to produce a bulk region of water in the centre of the channel [19]. Periodical conditions are applied in the *x* and *z*-directions.

The mutual effect model between atoms uses Lennard-Jones (LJ) and Coulomb potentials as given by,

$$\text{U}(r_{ij})=4\varepsilon \left[{\left(\frac{\sigma }{r_{ij}}\right)}^{12}-{\left(\frac{\sigma }{r_{ij}}\right)}^{6}\right]+\frac{1}{4\pi \varepsilon_{0}}\sum_{i}^{a}\sum_{j}^{b}\frac{q_{i}q_{j}}{r_{ij}},$$ (1)

where *ε* denotes the potential well depth, which reflects the strength of mutual interaction between two atoms, and *σ* is the distance between atoms when the action potential is equal to zero; *ε*_0_ represents the dielectric constant of a vacuum, *q*_*i*_ and *q*_*j*_ are the point charges of atoms *i* and *j*, respectively, *r*_*ij*_ is the distance between atoms *i* and *j*. The mutual effect potential parameters between oxygen atoms are *ε*_*oo*_ = 0.1553 Kcal/mol and *σ*_*oo*_ = 3.166 Å. The LJ interaction between hydrogen atoms and other atoms is set to zero. The mass of the oxygen atom is 15.994 g/mol, and the mass of the hydrogen atom is 1.008 g/mol, respectively. The charge of each oxygen atom is -0.820*e*, and the charge of each hydrogen atom is 0.410*e*, respectively, where *e* is the proton charge. The characteristic length of LJ between atomic oxygen and the solid is *σ*_*oo*_ = 3.166 Å.

In the present work, the SPC/E model is adopted for the water molecule. The SHAKE algorithm is used to maintain a constant bond length and angle between water molecules. The interaction between the solid atoms is cancelled in the name of computational efficiency. In the present work, the wall is changed in a manner similar to that adopted elsewhere [22]. For both walls, the charge is evenly distributed on the innermost solid atoms. The upper wall has a positive charge and the lower wall has a negative charge, which allows the system to meet the requirement for neutrality of the simulation box, so that the system can be modelled accurately by the particle-particle particle-mesh (PPPM) algorithm. The surface CD considered in this work is *CD* = 0 μC/cm^2^ to 26.24 μC / cm^2^, which is similar to that used in previous studies. A cut-off distance of 1 nm is used in all LJ calculations and Coulomb potential analyses (the Coulomb potential is solved by use of the PPPM algorithm).

The condition for simulating Poiseuille flow is such that a constant acceleration is applied to each oxygen and hydrogen atom in the +*x-*direction. Among them, the Nosé-Hoover thermostat is set to 300 K during the simulation, and the density of water to 1000 kg/m^3^. To achieve a steady flow, after 10^6^ MD time-steps, another 10^6^ MD time-steps are used to apply a constant acceleration to the oxygen and hydrogen atoms. MD simulation uses the open source LAMMPS MD code [23] with time-step Δ*t* = 1 fs.

The Navier model is used to calculate slip length, *Ls* = *Vs*/*γ*, where *V*_*s*_ is the slip velocity at the water-solid interface and *γ* represents the shear rate at the water-solid interface. The position of the water-solid interface is placed at the bottom of the upper wall surface and the top of the lower wall surface of the model. To obtain the values of the parameters *V*_*s*_ and *γ*, we first obtain the velocity distribution in the centre of the channel through a parabolic function, thus obtaining a mathematical expression for the velocity and shear rate distributions. Finally, substitution of the position of the water-solid interface into the corresponding yielded values of *V*_*s*_ and *γ*.

## 3 Results and discussion

### 3.1 Density and velocity profiles

**Fig 2:**
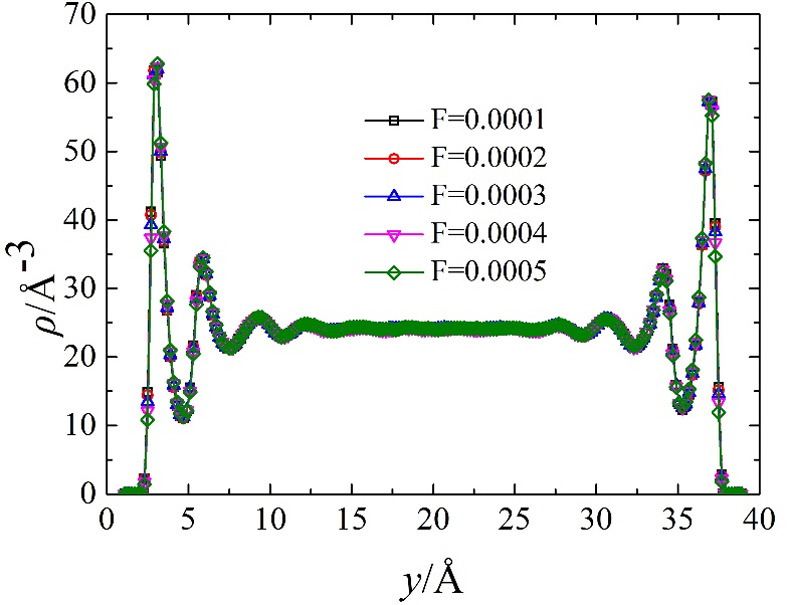
Electric field intensity and density distribution of driving force.

Fig 2 shows a representative density distribution under the interaction of different electric field strengths and driving forces: the density distribution presents a deep decaying oscillation near the two metal walls. In the centre of the channel, the water density is equal to the expected value. The first peak of the density distribution of adjacent walls is the so-called contact density, which is closely related to the slip length [23, 24]. Under the action of electric field strength and driving force, from the density distribution, the curves almost overlap, so under the magnitude of electric field and driving force, the density profiles are unaffected by the electric field and driving force. Water molecule density profiles are presented in Fig 2. There is strong fluid ordering evident across the whole channel.

In nanocomposite fluids, continuous fluid dynamics describe the velocity distribution in the centre of the channel well [25–29]. The solution of the Navier-Stokes equation for incompressible steady Poiseuille flow without slip BC is given by:

$$v_{x}(y)=\frac{\rho F_{x}H^{2}}{2\mu }[\frac{1}{4}-{\left(\frac{y}{H}-\frac{1}{2}\right)}^{2}],$$ (2)

where *H* is the channel height and *μ* denotes the fluid shear viscosity. However, Eq (2) can be corrected by use of the slip velocity at the water-solid interface [25–29].

**Fig 3:**
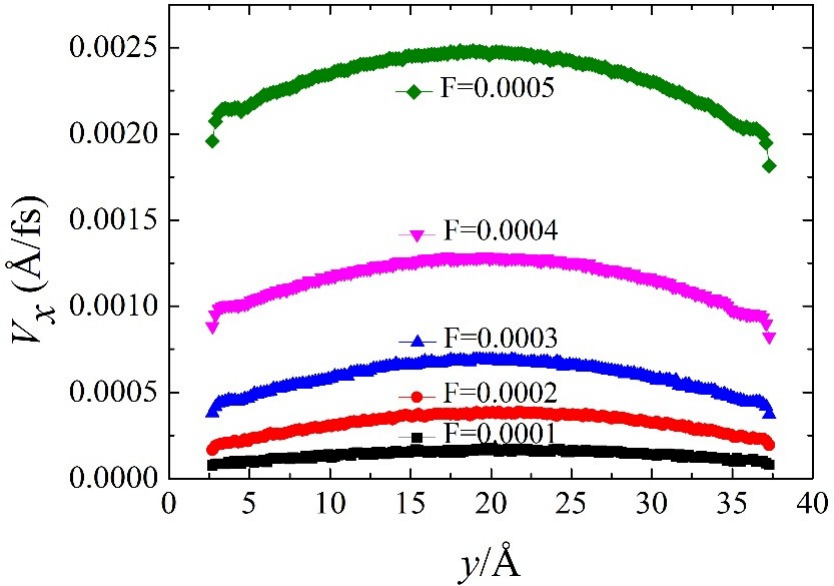
Electric field intensity and velocity distribution of driving force.

Fig 3 illustrates the typical velocity distribution of the interaction between the electric field strength and the driving force in a steady-state flow at selected values. As predicted by continuous fluid mechanics, the liquid water velocity at the centre of the channel is that predicted by a parabolic distribution [Eq (2)]: when the electric field strength is constant, the slip velocity increases with increasing driving force. For a given electric field strength, the increasing driving force generates a greater contact density: the larger driving force also increases the magnitude of the contact density for a given water-solid interaction energy. A correlation between the liquid water structure near the wall and the slip length will be examined in the next section.

### 3.2 The slip behaviour upon interaction of different electric field strengths and driving forces

**Fig 4:**
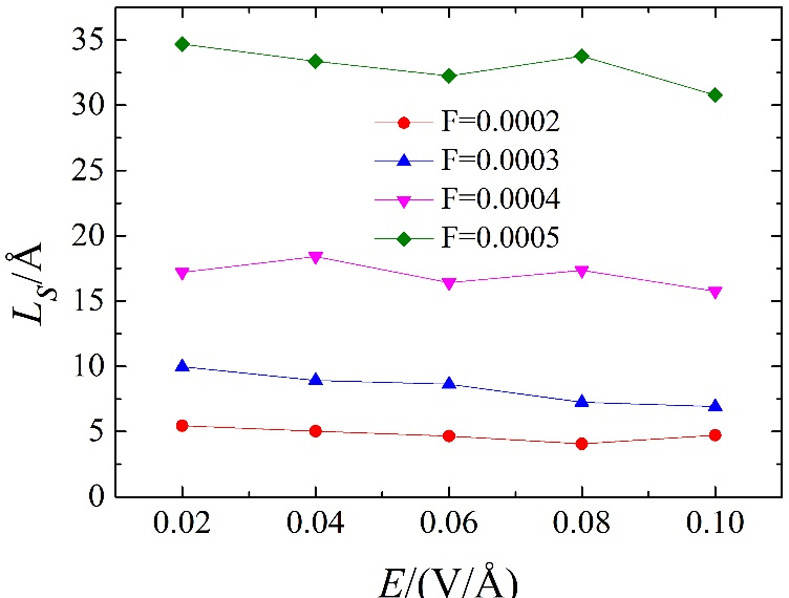
The slip length under the interaction of electric field strength and driving force.

For the electric field intensity of different surfaces, the slip length changes with the increase of the driving force (Fig 4). For comparison, Fig 4 also shows the neutral solid wall data. For a neutral solid wall, the slip length increases as the driving force increases. The relationship between slip length and driving force interaction is non-linear, and the correlation of slip length can be fitted with a quadratic function. When a driving force is applied, for a given electric field strength, the slip length is unchanged, which is consistent with the velocity profile shown in Fig 2. Under the same driving force, increasing the electric field strength has almost no effect on the slip behaviour. In addition, under the action of a small driving force, the slip length is significantly reduced. Under the action of the minimum driving force, the boundary condition (as applied to the water) becomes slip-free.

### 3.3 Analysis of slip behaviours from the perspective of the static structure factor

We found that the degree of slip at the water-solid interface is closely related to the amount of surface-induced order in the adjacent fluid layer. The in-plane static structure factor concept can be used to quantify the sensing structure of the metal wall as it affects the liquid water. The density distribution shows that there are several layers of liquid water near the metal wall, and the position of each layer of atoms is used to calculate the static structure factor in the plane. Therefore, *S*(k) is a quantitative measure of the in-plane order of each layer of water near the wall. The slip length is found to be inversely proportional to the size of the main peak of the static structure coefficient of the first water layer (FWL). The FWL is defined as the water molecules in the area between the wall and the first minimum in the density profile. The in-plane static structure factor is given by

$$S(k)=\frac{1}{N}{\left|\sum_{j}e^{ik\cdot r_{j}}\right|}^{2},$$ (3)

where **r**_*j*_ = (*x*_*j*_, *z*_*j*_) represents the two-dimensional position vector of the *j*-th atom, and the sum takes *N* atoms in the FWL. Here, **k** = (*k*_*x*_, *k*_*z*_) is the reciprocal vector parallel to the wall. In a limited system, the components of the vector *k* are confined to integer multiples of 2π/*L*, where *L* is the size of the system in the *x* and *z*-directions, therefore, the size of the system is inversely proportional to *k*_*x*_ and *k*_*z*_.

**Fig 5:**
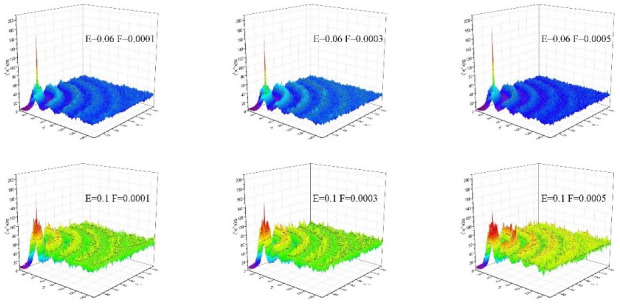
Examples of static structure factors of zero surface CD under different driving forces and electric field strengths.

The size of the system and the number of atoms determine the number of *S*(**G**_1_). In the present simulation, the size of the driving force determines the average number of fluid atoms in the FWL. Therefore, the size-independent number *S*(**G**_1_)/*S*(0), which exceeds 1 ns on average, is used to associate the water structure and sliding length [30]. We first give examples of static structure factors with the same electric field strength under different driving forces (Fig 5). Under the action of a strong electric field *E* = 0.06 and a strong driving force *F* = 0.0005, the static structure factor *S*(**G**_1_)/*S*(0) in the plane oscillates, is attenuated, and a peak appears at the lattice vector [31]. This sharp peak shows that the water has a certain degree of order, but the water molecules have not yet crystallised. The degree of order refers to the degree to which water molecules are arranged as a solid: the order of water molecules near the wall can be measured by the amplitude of the main peak of the static structure factor in the plane.

By comparison, in the case of the same electric field strength, we found that the smaller the driving force, the larger the main peak value of the static structure factor in the plane, and the higher the order of water molecules near the metal solid wall. The metal solid wall has a face-centred cubic structure, which shows that the metal solid atoms are arranged in a certain periodic manner, and a similar periodic potential field is generated on the metal solid wall [32]. It has been shown that the ordering within the water molecules near the wall is caused by the periodic potential generated by the walls [26, 33]. For a given electric field strength, a smaller driving force will have more ripple periodic potential than a larger driving force, and the periodic potential exerts a stronger binding force on the mobility of water molecules. In other words, the diffusion of water molecules becomes weaker leading to higher ordering, the larger the main peak of the in-plane static structure factor, the better the solid structure, the higher the degree of ordering. Therefore, the smaller the driving force, the smaller the slip length. When the driving force is increased to *F* = 0.0005, the periodic potential induced by the wall becomes smoother, resulting in a weaker restriction of the wall potential on water molecules, that is, the main peak value of the static structure factor in the plane becomes smaller, so the slip is greater.

**Fig 6:**
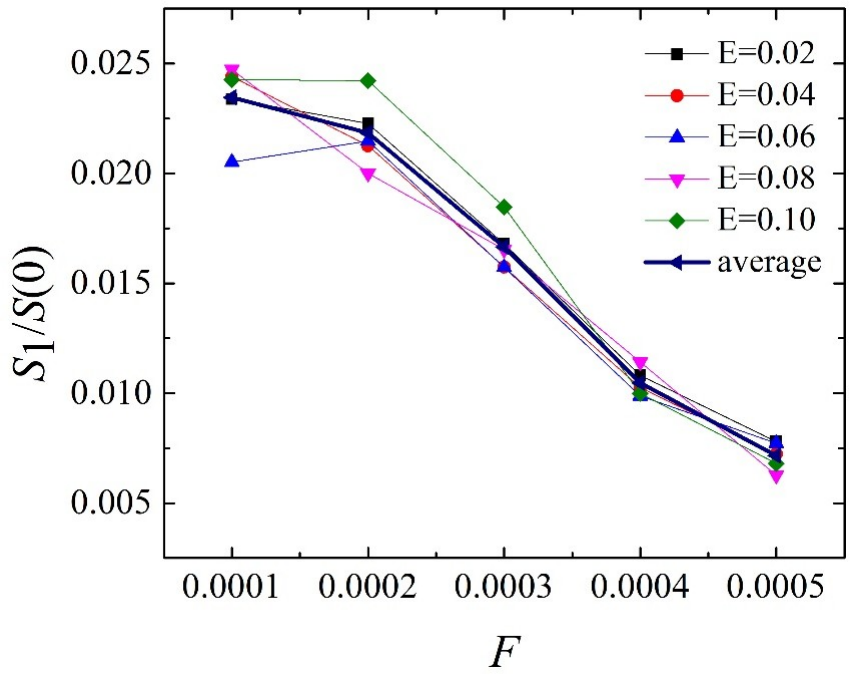
For the specified electric field strength, under different driving forces, the normalised main peak of the static structure in the plane, *S*(G1)/*S*(0).

Fig 6 shows the normalised main peak of the static structure factor in the plane under different driving forces for a specified electric field intensity. Fig 5 shows that, for a given electric field strength, the normalised main peak value of the in-plane static structure decreases as the driving force increases. It is consistent with the slip behaviour shown in Fig 4, therefore, as the driving force is increased, the slip length is increased, and a larger driving force produces greater slip.

It can be seen from Fig 6 that the non-linear relationship of the normalised main peak of the static structure factor in the driving force plane has become linear, and the relationship between the slip length and the driving force has changed in a similar manner. For a given driving force, for different electric field strengths, the normalised main peaks of the in-plane static structure differ little and the electric field strength has almost no effect on the slip behaviour. The main reason for this is that the larger the driving force, the smaller the normalised main peak of the static structure in the plane (the greater the slip length), corresponding to Fig 4. The slip length under different electric field strengths is almost unchanged under the same driving force. The detailed relationship between the sliding length and the main peak of the in-plane static structure is described next.

**Fig 7:**
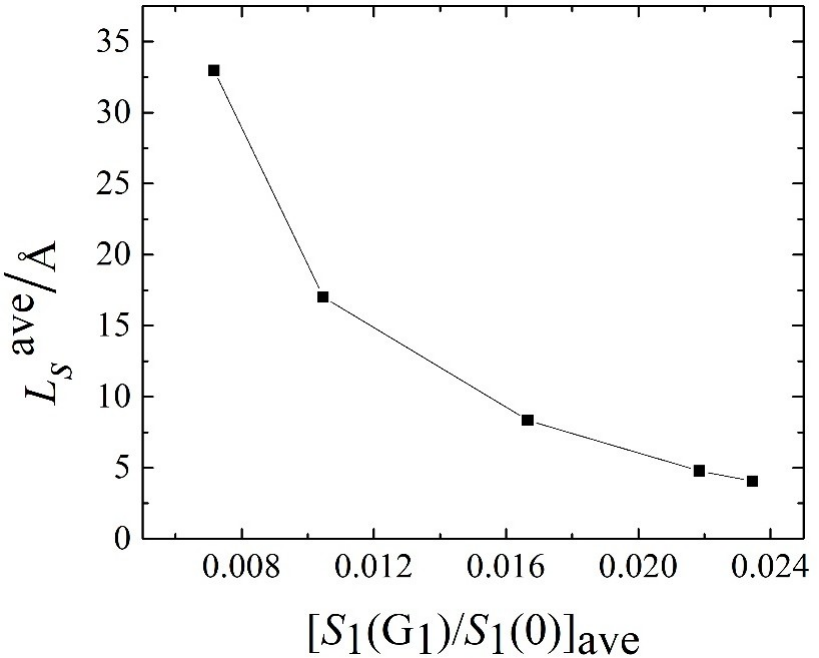
The behaviour of the average slip length, least squares, as a function of the normalised main peak of the in-plane structure coefficient, *S*(G_1_)/*S*(0).

Fig 7 shows the correlation between the average value of the liquid water structure factor evaluated at the first reciprocal lattice vector **G**_1_ and the average slip length. The normalised in-plane structure factor main peak is folded onto a non-linear plot to reflect different driving forces. We found that a similar proportional relationship exists on the simple fluid at the atomic level. This relationship is extended to the case of liquid water flowing over the metal surface.

## 4 Conclusion

Non-equilibrium MD simulation is used to study the influences of electric field strength and driving force on the sliding behaviour of liquid water on a metal surface. A constant acceleration is applied to produce a Poiseuille-like flow regime in which, the upper wall of the model is positively charged, and the lower wall is negatively charged.

The results show that the slip length increases as the driving force increases. From simulations, we found that under a given driving force, whether increasing or decreasing the electric field strength, the effect on the slip behaviour is negligible. The orderly structure of liquid water near the wall decreases as the driving force increases. The existence of the driving force reduces the orderly structure of the liquid water and reduces the degree of ordering, resulting in an increase in the slip length with increased driving force. The increase in driving force changes the dependence of the normalised main peak of the static structure factor on the driving force from a non-linear relationship to a linear relationship, directly effecting similar changes in the dependence of the slip length on the driving force. In addition to this, we also extended the relationship between the average slip length and the normalised main peak of the static structure factor from a simple liquid to water.

## Supporting information

S1 Data (ZIP)
